# Tuberculose laryngée isolée de l’adulte: localisation extra pulmonaire exceptionnelle (à propos d’un cas)

**DOI:** 10.11604/pamj.2022.43.9.21014

**Published:** 2022-09-06

**Authors:** Imen Mariem Abbassi, Mounira El Euch, Fatima Jaziri, Asma Kefi, Fethi Ben Hamida, Sami Turki, Khaoula Ben Abdelghani, Taieb Ben Abdallah

**Affiliations:** 1 Department of Internal Medicine, University of Tunis El Manar, Tunis, Tunisia,; 2 Research Laboratory of Kidney Diseases (LR00SP01), Charles Nicolle Hospital, Tunis, Tunisia

**Keywords:** Tuberculose, granulomatose, larynx, cas clinique, Tuberculosis, granulomatosis, larynx, case report

## Abstract

La tuberculose laryngée isolée est rare et parfois de diagnostic difficile. Elle représente la cause la plus fréquente de granulomatose laryngée. Nous rapportons le cas d´un homme âgé de 58 ans, sans antécédents pathologiques, hospitalisé pour une dyspnée laryngée paroxystique, dysphagie aux solides et dysphonie évoluant depuis 6 mois sans autres signes associés. L´examen laryngoscopique avait montré une formation polyploïde masquant l´étage glottique. L'examen histologique a révélé des granulomes épithélioïdes et giganto-cellulaires sans nécrose caséeuse. L´examen direct microscopique et la culture étaient négatifs. Le diagnostic de tuberculose laryngée isolée a été retenu devant l´endémicité de notre pays et l´absence d´autres arguments en faveur d´une autre granulomatose. Le traitement antituberculeux, associé à une corticothérapie orale, indiquée devant un important œdème des voies aériennes supérieures a permis une résolution des symptômes au bout de 40 jours de traitement.

## Abstract (en)

Isolated laryngeal tuberculosis is rare and sometimes difficult to diagnose. It is the most common cause of laryngeal granuloma. We here report the case of a 58-year-old man, with no particular past medical history, hospitalized due to paroxysmal laryngeal dyspnea, dysphagia to solid foods and dysphonia evolving for 6 months without other associated signs. Laryngoscopic examination showed polyploid formation masking the glottic floor. Histological examination revealed epithelioid and gigantocellular granuloma, without caseous necrosis. Direct microscopic examination and culture were negative. The diagnosis of isolated laryngeal tuberculosis was made based on the endemicity in our country and the absence of other arguments in favor of another type of granulomatosis. Anti-tuberculosis therapy, combined with oral corticosteroids, was indicated based on the presence of severe upper airway edema and symptoms were resolved after 40 days of treatment.

## Introduction

La tuberculose est une infection systémique fréquente dans le monde. En 2016, on recensait 10,4 millions de nouveaux cas (toutes formes confondues) de par le monde et 1,7 million de décès pouvant lui être attribués [1]. La Tunisie est un pays à endémicité intermédiaire avec une incidence enregistrée de 29/100000 habitants en 2017 [2]. Les formes extra pulmonaires sont de plus en plus diagnostiquées dont la localisation laryngée reste rare. En effet, la tuberculose laryngée (TL) représente 1% de toutes les localisations extra-thoraciques, et 46% au sein des voies aériennes supérieures [3]. Nous rapportons le cas d´un patient ayant une TL isolée découverte suite à une dyspnée laryngée, dysphonie et dysphagie aux solides.

## Patient et observation

**Information sur le patient:** un homme de 58 ans, originaire de Tozeur, n´ayant pas d´antécédents personnels, non tabagique ni éthylique, a consulté pour une dysphonie, des accès de dyspnée inspiratoire ainsi qu´une dysphagie aux solides évoluant depuis six mois, à début insidieux, sans notion d´altération de l´état général. Le patient a été vacciné par le Bacille de Calmette et Guérin (BCG) dans son enfance et il ne rapportait pas de notion de contage tuberculeux ni fièvre ni sueurs nocturnes.

**Examen clinique:** il n´avait pas montré d´adénopathies périphériques ni hépato-splénomégalie ni autre signe physique. L´examen à la naso-fibroscopie a mis en évidence une formation polyploïde masquant l´étage glottique prenant insertion sur l´aryténoïde gauche. A la laryngoscopie directe, on a noté un aspect boudiné et œdémateux du repli épiglottique gauche. Une première biopsie avait montré des remaniements fibro-inflammatoire non spécifiques. L´Imagerie par résonnance magnétique (IRM) cervicale a objectivé une hypertrophie du repli épiglottique plus importante à gauche, sténosant la lumière laryngée sus glottique et comblant le sinus piriforme, associée à une adénomégalie rétro-pharyngée droite. Une deuxième biopsie a été réalisée et a conclu à des lésions granulomateuses épithélioïdes et gigantocellulaires sans nécrose caséeuse. L´examen direct microscopique et la culture étaient négatifs.

**Résultats et suivi**: au bilan biologique, il n´y avait pas de syndrome inflammatoire, les globules blancs étaient à 7800 éléments/mm^3^, avec une formule leucocytaire normale. La radiographie du thorax n´avait pas montré de lésions parenchymateuses. L´Intradermoréaction (IDR) à la tuberculine était négative ainsi que la recherche de Bacille de Koch dans les crachats et les urines. Les sérologies VIH et syphilitique, virus Epstein-Barr (EBV), infection à cytomégalovirus (CMV) étaient négatives. Le taux de l´activité de l´enzyme de conversion était normal. Le bilan phosphocalcique sanguin et urinaire était sans anomalies. Les anticorps antinucléaires et anticytoplasmiques des polynucléaires étaient négatifs. La tomodensitométrie (TDM) du massif facial et thoraco-abdomino-pelvien n´a pas montré de signes en faveur de vascularite ni d´autres anomalies évocatrices d´une autre localisation de tuberculose. Le diagnostic de tuberculose laryngée isolée a été retenu devant l´endémicité de notre pays et l´absence d´autres arguments en faveur d´une autre granulomatose. Une quadrithérapie antituberculeuse a été démarrée. L´évolution clinique était favorable et la laryngoscopie refaite après 7 jours de traitement avait montré un œdème très important des voies aériennes supérieures sans retentissement clinique. Une corticothérapie générale a été initiée parallèlement, avec disparition de la dysphonie et de la dysphagie au bout de 40 jours de traitement.

**Consentement éclairé:** après des explications claires et détaillées, le patient a donné son consentement éclairé pour la réalisation de ce travail.

## Discussion

La TL est une localisation rare. Elle représente, généralement, une complication de tuberculose pulmonaire (TP). La plupart des patients avec une TL ont une TP active concomitante avec un taux d'expectorations positives de 90 à 95% [4]. Une TL isolée est par contre exceptionnelle. Malgré la rareté de cette entité, elle représente la principale étiologie de granulomatose laryngée [5], et survient principalement chez les adultes âgés de 40 à 60 ans comme l´illustre notre observation [6]. Quelques facteurs de risque ont été rapportés, comprenant l´absence de vaccination par le BCG, la malnutrition, la promiscuité, le syndrome d'immunodéficience acquise, l´immunosuppression et le tabagisme [7] qui n´étaient pas présents pour notre patient. Les signes généraux sont souvent discrets au cours des formes primitives [8], tel est le cas pour notre patient. Deux théories de contamination du larynx par le *Mycobacterium tuberculosis*, ont été proposées: une théorie bronchique déclarant que le larynx est infesté à travers l´arbre bronchique, et une théorie hématogène indiquant une infestation par voie hématogène à partir d´autres localisations tuberculeuses, autre que pulmonaire [9]. Notre patient n´avait pas d´atteinte pulmonaire associée, ce qui serait en faveur d´une infestation par voie hématogène par réactivation d´un foyer laryngé apparu suite à une primo-infection tuberculeuse. Chez notre patient, les signes fonctionnels révélateurs étaient une dysphagie, dysphonie et dyspnée laryngée paroxystique qui étaient rarement rapportés dans la littérature. Il s´agissait surtout d´un enrouement de la voix (80% à 100%) et d´odynophagie (50% à 67%) [10,11].

Ces symptômes sont aussi courants au cours des néoplasies du larynx. La dyspnée se voit généralement à un stade avancé de la maladie. Notre patient, bien qu´il fût asymptomatique au cours de son hospitalisation, avait un important œdème des voies aériennes supérieures, ayant pu mettre en jeu son pronostic vital. Les lésions constatées à la laryngoscopie suggèrent souvent un cancer du larynx, d´autant plus qu´il n´existe pas de preuve bactériologique de tuberculose laryngée [12,13]. En effet, la TL peut se manifester, macroscopiquement, par un œdème, une hyperémie, des ulcérations, un nodule ou une masse exophytique. Les cordes vocales sont le site le plus touché, suivies du pli ventriculaire, de l'épiglotte, de la région sous-glottique et de la commissure postérieure [10,14]. Elle doit être suspectée chez un homme présentant une laryngite chronique non spécifique évolutive. La biopsie laryngée avec étude anatomopathologique et bactériologique sont l´examen clé du diagnostic associé aux données anamnestiques et aux examens paracliniques qui peuvent rendre le diagnostic plus facile [8]. Bien que la preuve bactériologique était absente chez notre patient, un traitement antituberculeux d´épreuve a été initié aboutissant au rétablissement clinique et laryngoscopique au bout de 40 jours. L´amélioration rapide de la symptomatologie insiste sur l´importance de la rapidité d´instauration du traitement.

## Conclusion

La tuberculose laryngée est une affection rare. Elle doit être suspectée chez les sujets à risque. La biopsie et les prélèvements mycobactériologiques sont nécessaires pour le diagnostic permettant d´éliminer un cancer du larynx. D´autre part, il ne faut pas oublier d´éliminer les autres étiologies de granulomatoses laryngées. De ce fait devant une granulomatose isolée, un bilan lésionnel pourrait orienter le diagnostic final afin de pouvoir démarrer une thérapeutique adaptée à chaque pathologie et d´éviter des conduites abusives pouvant bouleverser le pronostic des patients.
